# Coinfection of *Cedecea lapagei* and *Aspergillus sydowii* detected in bronchoalveolar lavage fluid of a patient with pulmonary infection using metagenomic next−generation sequencing: A case report

**DOI:** 10.1016/j.heliyon.2024.e33130

**Published:** 2024-06-15

**Authors:** Yan Yang, Yingyue Zhao, Xiaotong Xi, Ran Ding, Lei Yang

**Affiliations:** a Panzhihua Central Hospital, Sichuan Province, China; b State Key Laboratory of Neurology and Oncology Drug Development (Jiangsu Simcere Pharmaceutical Co., Ltd.), Jiangsu Simcere Diagnostics Co., Ltd., China; c Nanjing Simcere Medical Laboratory Science Co., Ltd., China

**Keywords:** *Cedecea lapagei*, *Aspergillus sydowii*, Metagenomic next-generation sequencing, Pulmonary infection, Case report

## Abstract

**Background:**

*Cedecea lapagei* (*C. lapagei*), as a potential human pathogen, has been reported in limited cases of human infections in medical literature. However, the increasing frequency of isolating *Cedecea lapagei* from clinical specimens underscores its growing clinical significance that should not be underestimated. *Aspergillus sydowii* (*A. sydowii*), commonly isolated from various environments, serves as a pathogen of human cryptic aspergillosis. Clinical pathological changes caused by *A*. *sydowii* are not obvious, posing a significant challenge in clinical diagnosis. Consequently, metagenomic next-generation sequencing (mNGS) are required for precise differentiation and identification of pathogens.

**Case description:**

Here we present a case demonstrating successful treatment outcome in a patient with pulmonary infection caused by coinfection of *C. lapagei* and *A*. *sydowii*, as identified through metagenomic next-generation sequencing. The patient, a 50-year-old male, presented with worsening cough, sputum production, and hemoptysis. Metagenomic next-generation sequencing (mNGS) analysis of the bronchoalveolar lavage fluid (BALF) revealed the presence of both *C. lapagei* and *A*. *sydowii*. Subsequently, *C. lapagei* was also detected by culture in the same BALF sample, however while clinical fungal cultures and (1–3)-β-D glucan testing yielded negative results. Based on these findings along with imaging features and clinical symptoms of the patient, the final diagnosis was determined to be a co-infection of *C. lapagei* and *A. sydowii*.

**Conclusion:**

The clinical manifestations of human infections caused by *C. lapagei* are not specific; patients with cryptic aspergillosis may have been previously overlooked due to improper specimen selection or negative routine tests. Therefore, precise identification of pathogens is crucial. This case report highlights the value of mNGS in detecting *C. lapagei* and *A. sydowii* in BALF, enabling timely diagnosis with coinfections.

## 1 Introduction

Respiratory system infections pose a significant global challenge. Traditional microbial detection techniques often fall short in providing timely and accurate pathogen information. In contrast, metagenomic next-generation sequencing (mNGS) has the capability to detect a diverse range of pathogens in samples simultaneously. This ability aids in the swift and precise diagnosis of respiratory infections, laying the groundwork for personalized treatment plans [1]. The unbiased approach of mNGS enables not just infection diagnosis but also facilitates microbiome analysis, host response evaluation, and prediction of drug resistance giving it broad application potential in lower respiratory tract infections (LRIs) [2].

*Cedecea lapagei* (*C. lapagei*) was first discovered in 1977, but its potential pathogenicity remained unrecognized until 2006 when a case of environmentally acquired *C. lapagei* peritonitis was reported [3,4]. Subsequently, cases of *C. lapagei*-induced pneumonia have been reported in various countries including the United States, Korean, Brazilian, India, Vietnam, and Peru, these cases exhibited positive outcomes and favorable responses to antibiotics treatment [[5], [6], [7], [8], [9], [10], [11]]. Furthermore, *C. lapagei* has been identified as a causative agent for sinusitis, tissue infections, oral ulcers, traumatic wound infections and bacteremia following burns [[12], [13], [14], [15], [16]]. *Aspergillus sydowii*(*A. sydowii*) is commonly found in soil and marine environments, as well as on mouldy gypsum wall-board, dust and various foods [17,18]. Given its ubiquitous presence in the environment, humans are easily exposed to these microorganisms through direct contact such as skin contact, respiratory inhalation and dietary intake [17]. It has been reported in the literature that *A. sydowii* is an opportunistic pathogen capable of causing superficial skin infections, onychomycosis, otomycosis, and mycetoma, but there have been rare reports of cases where it caused respiratory infections in humans [[19], [20], [21], [22]].

The human respiratory tract serves as a portal for numerous microorganisms, and fungal spores can be inhaled and adhere to the respiratory tract. When the immune system is compromised and unable to eliminate them from the airways, invasive infections may occur, such as acute or chronic lung disease [23]. A study investigated the fungal diversity in exhaled breath condensate (EBC) samples obtained from healthy adult subjects. Among ten tested individuals (37.03 %), fungi were detected in their EBC samples, with *A. sydowii* being one of the most prevalent species identified [17]. Three participants who had detectable fungi in their EBC were diagnosed with respiratory system disorders, suggesting that *A. sydowii* could be a potential human pathogen [17]. Herein, we present a case of pulmonary infection caused by coinfection with *C*. *lapagei* and *A*. *sydowii* identified through mNGS. To our knowledge, this is the first reported instance of rapid detection of uncommon bacteria and rare fungi causing pneumonia using mNGS.

## 2 Case presentation

On May 30, 2023, a 50-year-old male was admitted to the Panzhihua Central Hospital in Sichuan Province due to severe cough and productive cough with bloody sputum. The patient had been experiencing productive cough without any clear etiology for the past year, frequently expectorating yellow purulent sputum and occasionally noticed blood-tinged sputum. A palpable mass was identified on the right side of his neck, however, the patient did not seek medical attention until recently when the symptoms worsened. Upon admission, he was diagnosed with a lung opacity requiring further investigation to determine its nature. Therefore, he was admitted to the hospital for further treatment.

The patient was born in their native place and has been residing locally for an extended period. The patient denied any history of contact with contaminated water and residing in epidemic areas. Denies history of chronic diseases such as coronary artery disease, diabetes, as well as infectious diseases like hepatitis and tuberculosis. There is no history of surgeries or traumas. The physical examination revealed the following findings: body temperature was recorded as 36.7 °C, pulse rate measured at 70/min, respiratory rate observed at 20 breath/min, and blood pressure measured as 114/73 mmHg. The patient presented with a well-developed physique, moderate nutrition status, intact mental state, and exhibited a chronically ill appearance. A firm and immobile nodule measuring 2cm in diameter was palpable beneath the right mandible. The chest displayed a barrel-shaped configuration without sternal elevation, diminished breath sounds bilaterally, widened spaces, symmetrical vocal fremitus, dullness upon percussion of the lungs, and moist rales were auscultated without pleural friction rub.

The laboratory test revealed a white blood cell count of 4.69 × 10^9/L, lymphocyte count of 1.01 × 10^9/L, neutrophil count of 3.11 × 10^9/L, procalcitonin level of 0.036 ng/mL, blood sodium concentration of 141.3 mmol/L, blood potassium concentration of 4.02 mmol/L, and blood chloride concentration of 107.9 mmol/L. A chest Computer tomography (CT) scan indicated a cavity in the right upper lobe with slightly high-density lesions, bronchiectasis accompanied by inflammatory lesions, scattered fibrotic lesions, and patchy shadows observed in both upper lobes of the lungs. Following clinical assessment, it was decided to initiate empirical treatment with cefotaxime sodium 2.0g IV every 12 hours for suspected bacterial infection, with close monitoring of efficacy and adverse reactions. Simultaneously taking anning capsules orally to alleviate cough symptoms.

**Fig. 1:**
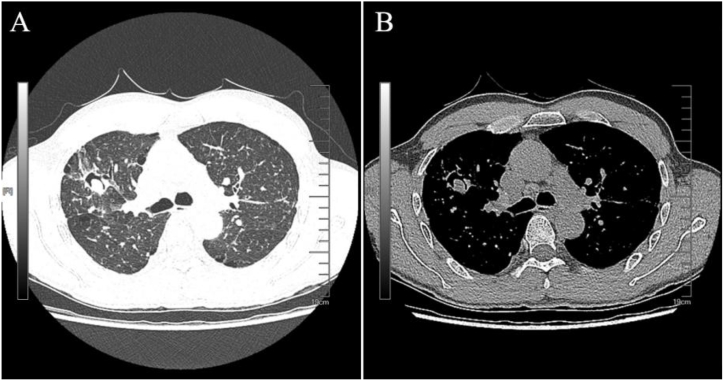
Chest CT scan.

On May 31, a chest CT scan with contrast enhancement and three-dimensional reconstruction revealed a thin-walled cavity measuring approximately 2.2 cm diameter in the apical segment of the right upper lobe. There was a slightly hyperdense lesion with nodular appearance and an “air crescent sign” can be observed inside the cavity, which remained unchanged following contrast enhancement (Fig. 1). The lesion demonstrated local communication with the bronchus. Additionally, multiple nodular hyperdense opacities measuring approximately 0.5 cm were observed bilaterally within the lungs, accompanied by scattered linear hyperdensities. The mediastinum exhibited central positioning along with multiple mildly enlarged. *Mycobacterium tuberculosis* Gene X-pert MTB/RIF test was performed using bronchoalveolar lavage fluid (BALF), with the result being negative. A neck CT scan of the neck revealed a high-density lesion in the right submandibular region with enlargement and blurred margins of the right submandibular gland, suggestive of possible intraductal calculus in the submandibular gland duct. Other differential diagnoses were not excluded, and follow-up imaging was recommended after consultation with the oral and maxillofacial department.

After carefully examining the aforementioned lung imaging results, a suspicion of fungal infection, potentially *Aspergillus*, emerged. Further targeted auxiliary examinations were conducted. A fungal (1,3)-β-D-glucan assay was performed on the blood sample obtained upon admission, yielding a negative result. Additionally, sputum and bronchoalveolar lavage fluid (BALF) were collected for fungal culture. Due to the time-consuming nature of fungal culture, the collected BALF sample was thoroughly mixed and divided into two aliquots, with one aliquot sent for mNGS testing. The sample was subjected to the laboratory on June 1st. DNA was isolated adhering to the guidelines provided by the manufacturer utilizing the TIANamp Micro DNA Kit. Subsequently, a DNA library was constructed with the Hieff NGS®OnePotTM II DNA Library Preparation Kit. Qualified libraries underwent sequencing via the NextSeq 550 system. After sequencing, adaptors and low-quality sequences were eliminated, while the removal of human DNA was achieved through alignment against the human reference database. The remaining sequences were compared with the microbial genome database (NCBI; https://www.ncbi.nlm.nih.gov/genome) to identify potential pathogens. On June 2, mNGS results of the pulmonary alveolar sample showed positive detection of bacteria, fungi, and viruses (Table 1). Among the detected bacteria, Haemophilus influenzae, *Klebsiella pneumoniae*, and Moraxella catarrhalis are common pathogens causing respiratory infections, with conventional antibiotic therapy covering these pathogens adequately. Considering the patient's imaging findings and clinical symptoms, viral infection is currently not considered. Therefore, human herpesvirus 7 detected in the viral list is not taken into consideration. Of concern are the detections of *C. lapagei* and *A. sydowii*. The sequencing results revealed high abundance of *C. lapagei* (1744405 specific reads) and *A. sydowii* (990 specific reads). On the same day (June 2), *C*. *lapagei* was cultured and subjected to drug sensitivity testing, which demonstrated susceptibility to cefuroxime, cefotiam, cefotetan, cefepime, piperacillin/tazobactam, compound neomycin, furantoin, levofloxacin, cycloserine, tylosin, gentamicin, amikacin, ertapenem, amoxicillin, and cefoperazone but resistant to cefazolin and ampicillin-sulbactam. Evidence strongly supports infection by *C. lapagei*. Based on results of bacterial culture and mNGS, considering the sensitivity of *C. lapagei* to third-generation cephalosporins, ceftriaxone was continued for treatment.

**Table 1:** Pathogenic organisms detected in BALF by mNGS.

Superkingdom	Genus			Species	
	genus	genus_reads	relative_abundance	species	species_reads
Bacteria	*Cedecea*	1,763,025	80.64 %	*Cedecea lapagei*	1,744,405
	*Haemophilus*	26,909	1.23 %	*Haemophilus influenzae*	25,593
	*Moraxella*	12,034	0.55 %	*Moraxella catarrhalis*	11,409
	*Klebsiella*	4,423	0.20 %	*Klebsiella pneumoniae*	2,784
Fungi	*Aspergillus*	1,327	24.08 %	*Aspergillus sydowii*	990
DNA-virus	–	–	–	Human betaherpesvirus 7	36

**Fig. 2:**
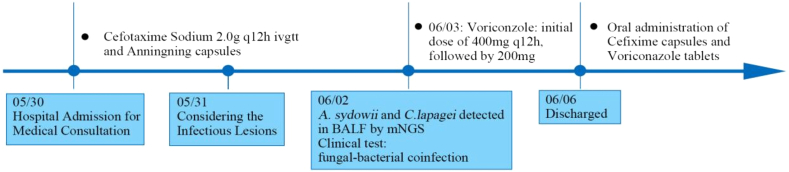
The treatment timeline of the patient.

The physician observed typical changes indicative of *Aspergillus* infection in the patient's pulmonary imaging. Following further consideration of factors such as the patient's pulmonary baseline and immune status, the clinical diagnosis indicates that the detected *A. sydowii* by mNGS is also responsible for the patient's condition. Thus, pulmonary aspergillosis infection is established. Voriconazole was added on June 3rd (initial dose of 400mg q12h, followed by 200mg q12h). Three days after medication initiation, the patient's symptoms significantly improved. The patient was discharged on June 6th. During the treatment period, there were no adverse events or unexpected incidents. Following discharge, the patient continued with oral administration of cefixime capsules and voriconazole tablets. On June 15th, the patient returned for follow-up and underwent chest CT scan with three-dimensional reconstruction, which showed stable imaging findings. However, clinical symptoms of cough, sputum production, and hemoptysis had significantly improved. Clinical improvement preceded imaging improvement, indicating a slight delay in radiological response compared to clinical symptoms. Please refer to Fig. 2 for a comprehensive overview of the diagnostic and treatment process.

## 3 Discussion

As infrequent human pathogens, *C*. *lapagei* and *A*.*s sydowii* have only been reported in a limited number of clinical cases to date. A search restricted to human studies in the PubMed database yielded a total of 13 cases of *C*. *lapagei*, with pneumonia being the predominant manifestation (6/13, Table 2). In a case study published in 2008 by Yetkin G. et al. from India indicated that a 38-year-old male patient with a chronic obstructive pulmonary disease and subarachnoid hemorrhage that developed pneumonia caused by *C*. *lapagei*, apparently due to secretion aspiration from the upper respiratory tract; this represented the first documented instance of *C*. *lapagei* pneumonia [5]. In 2013, Lopez LA et al. reported a case of severe pneumonia resulting from the co-infection of *C*. *lapagei* with other nosocomial pathogens in a patient diagnosed with acute promyelocytic leukemia in the United States [6]. Hong SK et al. isolated *C. lapagei* from sputum smear in a 76-year-old male with a history of chronic obstructive pulmonary disease from Korea in 2015 [7]. The only similarity between their reported case and ours was that the organism isolated by Hong SK et al. was also sensitive to piperacillin, cefotaxime, ceftazidime, cefepime, and ciprofloxacin. Similar to our case, this patient exhibited improvement after one week of treatment with cefpodoxime. No pathogens were detected in subsequent respiratory cultures, leading to the patient's discharge. Kury CMH et al. from Brazil and Ramaswamy VV et al. from India respectively published case reports on neonatal pneumonia and septicemia caused by *C*. *lapagei* in 2017 and 2019 [8,9]. In 2020, Hai PD et al. reported a case of pneumonia and septic shock caused by *C*. *lapagei* in a 38-year-old male in Vietnam. Notably, the majority of documented cases have been linked to immunodeficiency [10]. The infections caused by *A*. *sydowii* primarily manifest as superficial conditions, such as skin, hair, and nail infections, with only rare instances of invasive infections reported [19,20]. Currently, there have been no documented cases of involving both pathogens in humans.

**Table 2:** A comparison of the clinical detection methods, antibiotic sensitivity and outcomes of the seven cases of *Cedecea lapagei* pneumonia.

Author, country	Age of presentation, gender	Detection method	Antibiotic sensitivity	Outcome
Yetkin G et al., 2008, India	38-year-old, male	Direct microscopic examination, balf	Sensitive to meropenem and amikacin	died due to the subarachnoid hemorrhage
Lopez LA et al., 2013, America	34-year-old, male	cultures of sputum	Sensitive to tigecycline. Resistant to amikacin, ampicillin, aztreonam, cefazolin, cefepime, ceftriaxone, ciprofloxacin, ertapenem, imipenem, meropenem, moxifloxacin, nitrofurantoin, piperacillin/tazobactam and trimethoprim/sulfamethoxazole.	Discharged
Hong SK et al., 2015, Korean	76-year-old, male	smear of the sputum sample	Sensitive to piperacillin, cefotaxime, ceftazidime, cefepime, imipenem, amikacin, ciprofloxacin, tetracycline, and trimethoprim-sulfamethoxazole. Resistant to amoxicillin-clavulanic acid and cefoxitin.	Discharged
Kury CMH et al., 2017, Brazil	Day 110	Cultures of blood and tracheal aspirates	Sensitive to piperacillin, imipenem, amikacin, gentamycin, ciprofloxacin, levofloxacin, meropenem and trimethoprim-sulfamethoxazole. Resistance pattern not mentioned.	Not mentioned
Ramaswamy VV et al., 2019, India	Day 49	Culture of blood	Sensitive to piperacillin, piperacillin-tazobactam, trimethoprim- sulfamethoxazole, ciprofloxacin and levofloxacin. Resistant to meropenem, colistin, amikacin, gentamycin and ceftazidime.	Discharged
Hai PD et al., 2020, Vietnam	38-year-old, male	cultures of sputum	Sensitive to ampicillin/sulbactam, tigecycline, gentamicin and tobramycin. Resistant to amikacin, aztreonam, cefazolin, cefepime, ceftriaxone, ciprofloxacin, ertapenem, imipenem, meropenem, moxifloxacin, nitrofurantoin and colistin	Discharged
Present case	50-year-old, male	cultures of BALF + mNGS	Sensitive to cefuroxime, cefotiam, cefotetan, piperacillin/tazobactam, cefepime, compound neomycin, furantoin, levofloxacin, cycloserine, tylosin, gentamicin, amikacin, ertapenem, amoxicillin, and cefoperazone. Resistant to cefazolin and ampicillin-sulbactam.	Discharged

After admission, the patient underwent comprehensive baseline examinations, and imaging results suggested the possibility of pulmonary *Aspergillus* involvement. To further confirm the responsible pathogens, BALF samples and blood samples were collected for culture. The blood culture results were negative, while both the BALF culture and concurrent mNGS testing indicated the presence of *C*. *lapagei*. Considering cultivation as the gold standard and mNGS as a supplementary tool, clinically, evidence strongly supports infection by *C. lapagei*. Additionally, noteworthy is the detection of *A. sydowii* by mNGS, which correlates closely with the patient's imaging findings. Although *Aspergillus* was never cultured using traditional methods, considering the patient's clinical presentation—poor pulmonary condition, susceptibility, and typical radiological changes suggestive of aspergillosis—the clinical decision ultimately relies on mNGS results, confirming the diagnosis of pulmonary aspergillosis. Based on the BALF culture results and mNGS findings, a final diagnosis of concurrent infection was made. The patient exhibited improvement and was discharged after receiving targeted treatment with voriconazole (200mg q12h) and ceftaroline (100mg BID).

Regarding the source of infection, neither *C*. *lapagei* nor *A*. *sydowii* are constituents of the normal human skin microbiota. However, both pathogens exhibit wide environmental prevalence, thereby substantiating the plausibility of considering the patient's living the most probable origin of infection in our case. The patient resided in a village in Yunnan Province, China, characterized by warm and humid climate during the months of May to June. Seasonal variations have been reported in fungal loads within, potentially leading to increased fungal presence during this period [24]. Furthermore, strong winds can facilitate the dispersal of fungal spores or bacterial particles. Considering the patient's prolonged respiratory infection symptoms spanning over a year and compromised lung condition, inhalation would render them more susceptible to infection. Further support from additional reports is needed for the mentioned infectious source in this case report.

In summary, we present a case of pulmonary infection caused by a rare combination of bacteria and fungi, with favorable clinical outcomes and prognosis. Notably, this is the first reported instance of *C*. *lapagei* identified using mNGS technology. These results underscore the diagnostic and therapeutic advantages of mNGS of coinfections and its potential benefits for patients afflicted with such infections.

## Consent for publication

We have obtained written informed consent from the patient for the publication of this case report, any accompanying data and images.

## Data availability statement

In this study, all relevant data supporting the findings of this research are included within the article. Additional data that is not included in the article is available from the corresponding author upon reasonable request.

## Ethics declarations

All participants/patients (or their proxies/legal guardians) provided informed consent to participate in the study.

## CRediT authorship contribution statement

**Yan Yang:** Writing – review & editing, Investigation. **Yingyue Zhao:** Writing – original draft, Investigation. **Xiaotong Xi:** Writing – review & editing, Supervision. **Ran Ding:** Writing – review & editing, Supervision, Resources. **Lei Yang:** Writing – review & editing, Supervision, Resources.

## Declaration of competing interest

The authors declare that they have no known competing financial interests or personal relationships that could have appeared to influence the work reported in this paper.
